# Retraction: Linc00662 promotes tumorigenesis and progression by regulating miR-497-5p/AVL9 axis in colorectal cancer

**DOI:** 10.3389/fgene.2026.1784270

**Published:** 2026-01-16

**Authors:** Frontiers Editorial Office

The journal retracts the 24 January 2020 article cited above.

Following publication, concerns were raised regarding the integrity of the images in the published figures. The authors failed to provide a satisfactory explanation during the investigation, which was conducted in accordance with Frontiers' policies.

This retraction was approved by the Chief Editors of Frontiers in Genetics and the Chief Executive Editor of Frontiers. The authors received a communication regarding the retraction and had a chance to respond. This communication has been recorded by the publisher.

Frontiers would like to thank the concerned reader who contacted us regarding the published article.

